# Long-Term Use of Probiotics *Lactobacillus* and *Bifidobacterium* Has a Prophylactic Effect on the Occurrence and Severity of Pouchitis: A Randomized Prospective Study

**DOI:** 10.1155/2014/208064

**Published:** 2014-01-21

**Authors:** Banasiewicz Tomasz, Stojcev Zoran, Walkowiak Jarosław, Marciniak Ryszard, Grochowalski Marcin, Burdyński Robert, Krokowicz Piotr, Krokowicz Lukasz, Paszkowski Jacek, Gronek Piotr, Pyda Przemysław, Drews Michał

**Affiliations:** ^1^Department of General Surgery, Oncologic Gastroenterologic Surgery and Plastic Surgery, Poznan University of Medical Sciences, Ulica Przybyszewskiego 49, 60-355 Poznań, Poland; ^2^Regional Specialist Hospital, Department of General, Vascular and Oncologic Surgery, 76-200 Slupsk, Poland; ^3^Department of Oncologic Surgery, Gdansk Medical University, 80-210 Gdansk, Poland; ^4^I Chair of Pediatrics, Department of Gastroenterology and Metabolism, Poznan University of Medical Sciences, 60-572 Poznań, Poland; ^5^Department of General and Colorectal Surgery, Poznan University of Medical Sciences, 61-285 Poznań, Poland; ^6^Department of Physiology, University School of Physical Education, 61-871 Poznań, Poland

## Abstract

*Aim*. The aim of the study was to assess the impact of the long-term use of the composite probiotics in patients after restorative proctocolectomy. *Method*. Forty-three patients (20 females and 23 males, aged 21 to 68 years) after restorative proctocolectomy were included in the study. After randomization patients were divided into placebo group and treatment group with oral intake of probiotic containing *Lactobacillus acidophilus*, *Lactobacillus delbrueckii subsp. bulgaricus*, and *Bifidobacterium bifidus*. Patients were investigated during initial visit and during final visit after 9 months. All patients were subjected to standard clinical and endoscopic examination with microscopic study of the specimens. Concentrations of calprotectin and pyruvate kinase isoenzyme M2-PK were determined in all cases. *Results*. The average severity of pouchitis and the number of patients with pouchitis significantly decrease after 9 months of the probiotic taking. The concentrations of calprotectin and pyruvate kinase isoenzyme M2-PK significantly decreased after the therapy. *Conclusions*. Nine months of the probiotic treatment (*Lactobacillus acidophilus*, *Lactobacillus delbrueckii subsp. bulgaricus*, and *Bifidobacterium bifidus*) reduced the number of patients with pouchitis, decreased the PDAI score, and also decreased the fecal pyruvate kinase and calprotectin. The long-term probiotics use is safe and well accepted and can be an effective method of the pouchitis prevention.

## 1. Introduction

The Western human diet contains several thousand times less bacteria than preindustrialized diets, mainly due to improved hygiene and nutrition and the use of processed and sterile foods which contain artificial sweeteners and preservatives, rather than fresh fruits and vegetables [1]. Decreased concentration of the gut microbiome or dysbiosis may be implicated in gastrointestinal disorders including diarrheal diseases, ulcerative colitis, inflammatory bowel diseases, and life style diseases [2].

The use of probiotics in the treatment of the different GI tract diseases seems to be still more popular. Pouchitis—inflammation of the intestinal mucosa of the small intestine—is a common complication of restorative proctocolectomy. The data suggest positive role of the probiotics, especially in prevention of recurrence [3]. The randomized controlled trials using probiotics in patients with different inflammatory bowel diseases are required for probiotics to become mainstream therapy [4, 5].

The aim of the study was to assess the impact of the long-term use of the composite probiotic (*Lactobacillus acidophilus*, *Lactobacillus delbrueckii subsp. bulgaricus*, and *Bifidobacterium bifidus*) in patients after restorative proctocolectomy.

## 2. Material and Methods

Forty-three patients (20 females and 23 males, aged 21 to 68 years) after restorative proctocolectomy, operated on for ulcerative colitis (UC) and familial adenomatous polyposis (FAP) and admitted to outpatient clinic according to prescheduled followup and without acute signs of the pouchitis or other GI tract severe disorders, were included in the study. Patients were investigated during initial visit and during final visit after 9 months. During the followup 3 patients withdrew from the study—1 patient in placebo group and 2 in probiotic group. In all patients clinical, endoscopical, and histopathological examinations were done during both visits. Before the initial visit, during phone registration, patients were informed about stool samples collection day before or in the day of the visit. Samples taken more than 2 hours before the visit were stored in +4°C (refrigerator). The stool samples were also collected with the same rules before final visit. After investigation patients were randomized into two groups: placebo and probiotic groups. The study was performed in accordance with the Helsinki declaration and the regulations of the Ethics Committee of Poznan University of Medical Sciences.

### 2.1. Pouchitis Disease Activity Index (PDAI)

Modified Pouch Disease Activity Index (PDAI) was determined in all IPAA patients, including clinical, endoscopic, and histopathological (Moskowitz criteria) parameters [6]. Pouchitis was recognized, if the total points were ≥7.

### 2.2. Pyruvate Kinase and Calprotectin Level

Each participant (both patients and healthy controls) provided 3 samples of stool, which underwent spectrophotometric assessment of levels of calprotectin (Immundiagnostik AG, Bensheim, Germany) [7] and pyruvate kinase isoenzyme M2-PK (Schebo-Biotech, Giessen, Germany), after double reaction with monoclonal antibodies binding with specific epitopes of the enzymes (enzyme-linked immunosorbent assay, ELISA) [8]. Mean values from 3 measurements were calculated and used in further analyses.

### 2.3. Probiotic Therapy

The composite probiotic Trilac (Allergon AB, Angelholm, Sweden) was used in the investigation. Each capsule contained 0,6 × 10^9^ lyophilized *Lactobacillus acidophilus*, 0,4 × 10^9^ lyophilized *Lactobacillus delbrueckii subsp. bulgaricus,* and 0,6 × 10^9^ lyophilized *Bifidobacterium bifidus.* Patients in the probiotic group took Trilac in the doses of 3 × 2 capsules daily during first month and 2 × 1 capsule during next months. Visual analogue placebo capsules were administered in the same dose.

### 2.4. Statistical Analysis

Statistical analysis was performed using Statistica (Statsoft version 6.0). The statistical differences between groups were calculated using the Mann-Whitney test (nonpaired data).

## 3. Results

During the treatment good tolerance of the used probiotic was observed. There were no side effects and complications in probiotic, as well as in placebo groups. All 3 patients, who did not finish the study (1 patient in placebo group and 2 in probiotic group) withdrew from the investigation due to noninvestigation related reasons (1 person accident, 2 persons emigration). The probiotic group (*n* = 19) and the control group (*n* = 21) did not differ significantly within age, time after surgery, number of patients with pouchitis, average PDAI, M2-PK level, and calprotectin level in the stool before the study.

After 9 months of the study the number of the patients with pouchitis, average PDAI, average M2-PK, and calprotectin level in the placebo group has not changed. In the probiotic group significantly lower numbers of the patients with pouchitis and lower average PDAI were observed, as well as the significant decreases of the M2-PK and calprotectin level in the stool. Average PDAI in probiotic treated group was 6.28 points before therapy and 4,43 points after therapy, with statistically important differences (*P* = 0.000342). The M2-PK average level was 92.3 U/mL in probiotic group before therapy and 45.5 U/mL after therapy, with statistically important differences (*P* = 0.000322). The same tendency was observed in the calprotectin level—65.8 mg/mL in probiotic group before therapy and 30.1 mg/mL in the same group after therapy (*P* = 0.000236). Decrease of the average PDAI, the M2-PK, and calprotectin level means decreasing of the inflammation of the pouch mucosa.

Exact values reflecting the changes in the pouchitis frequency, PDAI, M2-PK, and calprotectin level before and after the study are presented in Table 1.

## 4. Discussion

Probiotics are popular method of the treatment of different gastrointestinal pathologies, mostly inflammatory. The main indications are irritable bowel syndrome and pouchitis. Probiotics are prescribed or recommended much more by the gastroenterologist as by the surgeons [9]. Probiotics are an option for recurrent and relapsing antibiotic sensitive pouchitis; this suggests potential for benefit in select patients, but concerns remain about proof from trials [10].

Information about the effectiveness of such treatment is not clear. Given the limited number of lines of evidence of probiotics efficacy from controlled trials and the many unanswered questions on probiotic treatment, only prescribing probiotic treatment for mild inflammation of the intestinal mucosa and as prophylaxis of the inflammation is recommended [11]. The short-term probiotics therapy has limited effectiveness [12].

In the probiotics treatment of pouchitis lasting for 2 months no significant differences of pouchitis process activity using PDAI scale were detected between probiotic and placebo intakes; however, significant decrease of bowel openings per day was observed in the probiotic group, as well as the subjective well-being improvement [13].

The benefits of the probiotics therapy can be strain specific [14]. The very popular and commonly used probiotic is VSL number 3, whose effectiveness has been confirmed in clinical [15], experimental [16], and computer controlled [17] models. Another popular source of probiotics is probiotic containing yoghurt and drinks, very popular supplements recommended for patients among surgeons [9].

In the presented study the composite probiotic Trilac, containing *Lactobacillus acidophilus*, *Lactobacillus delbrueckii subsp. bulgaricus,* and *Bifidobacterium bifidus* was used. There are a lot of studies that confirmed positive role of the probiotics *Lactobacillus* and *Bifidobacterium* in gastrointestinal disorders and gut microbiota [18–21].

In our study the average severity of pouchitis measured with the PDAI score and the number of patients with pouchitis significantly decrease after 9 months of the probiotic taking. Also more objective parameters, as the fecal markers, confirmed the positive role of the probiotic in limitation of the inflammation in the intestinal pouch mucosa. High efficacy of the probiotic therapy especially in the pouchitis patients is consistent with the observations of other authors [22]. This therapy was very well accepted by the patients, no side effects were observed, and the tolerance of the probiotic was high. In our opinion this method of the prevention of the pouchitis is very important, because the treatment of pouchitis is still not well established and in some cases complicated.

The other finding of the study is the confirmation of the usefulness of the noninvasive fecal markers in diagnosis and monitoring of the pouchitis. The used pyruvate kinase isoenzyme M2-PK in the study was first described as the marker of the pouchitis by Walkowiak et al. [8] and confirmed in the next studies [23]. Also fecal calprotectin is accepted tool for the detection and definition of the severity of the pouchitis [24]. Fecal markers are commonly accepted and adjunctive tools in overall evaluation of patients with inflammatory disease, as pouchitis, to monitor disease activity and modification of the treatment. This noninvasive method is much more safe and accepted than endoscopy and can help guide management in a more cost-effective manner [25].

In presented study correlation between fecal M2 pyruvate kinase and calprotectin correlated significantly with the severity of the pouchitis described in the PDAI score, as well as with the number of patients with recognized pouchitis.

## 5. Conclusions

Nine months of the probiotic treatment (*Lactobacillus acidophilus*, *Lactobacillus delbrueckii subsp. bulgaricus,* and *Bifidobacterium bifidus*) reduced the number of patients with pouchitis, decreased the average PDAI score, and also decreased the fecal pyruvate kinase and calprotectin. The long-term probiotics use is safe and well accepted and can be an effective method of pouchitis prevention.

## Figures and Tables

**Table 1:** Characteristics of the investigated group, pouchitis disease activity index (PDAI), and level of the pyruvate kinase (M2-PK) and calprotectin in the placebo and treatment group. Observation time—9 months, with asterisk (*) signing the data with statistically significant differences.

Patients	Placebo group before treatment	Placebo group after treatment	Probiotic group before treatment	Probiotic group after treatment
Number of patients	22	21	21	19
Number of patients with pouchitis (PDAI ≥ 7 pts)	7	8	7	3
Age of patients (years)	41.2	41.5	38.6	38.4
Time from operation (years)	9.2	9.1	8.9	8.9
PDAI	5.97	5.72	6.28*	4.43*
*P* value	*P* = 0.115861		*P* = 0.000342	
M2-PK (U/mL)	87.4	90.1	92.3*	45.5*
*P* value	*P* = 0.877654		*P* = 0.000322	
Calprotectin (mg/L)	57.5	55.8	65.8*	30.1*
*P* value	*P* = 0.107476		*P* = 0.000236	
